# A Review of Electrochemical Sensors for the Detection of Glycated Hemoglobin

**DOI:** 10.3390/bios12040221

**Published:** 2022-04-08

**Authors:** Zhikun Zhan, Yang Li, Yuliang Zhao, Hongyu Zhang, Zhen Wang, Boya Fu, Wen Jung Li

**Affiliations:** 1 School of Computer and Communication Engineering, Northeastern University at Qinhuangdao, Qinhuangdao 066004, China; zkzhan@ysu.edu.cn; 2 Key Laboratory of Intelligent Rehabilitation and Neuromodulation of Hebei Province, School of Electrical Engineering, Yanshan University, Qinhuangdao 066004, China; liyangysu@163.com (Y.L.); wz13230303639@163.com (Z.W.); 18830491838@163.com (B.F.); 3 School of Control Engineering, Northeastern University at Qinhuangdao, Qinhuangdao 066004, China; 4 Department of Mechanical Engineering, City University of Hong Kong, Kowloon, Hong Kong 999077, China; hyzhang42-c@my.cityu.edu.hk

**Keywords:** electrochemical sensor, HbA1c sensor, fructosyl valine sensor, diabetes, cyclic voltammetry, electrochemical impedance spectroscopy

## Abstract

Glycated hemoglobin (HbA1c) is the gold standard for measuring glucose levels in the diagnosis of diabetes due to the excellent stability and reliability of this biomarker. HbA1c is a stable glycated protein formed by the reaction of glucose with hemoglobin (Hb) in red blood cells, which reflects average glucose levels over a period of two to three months without suffering from the disturbance of the outside environment. A number of simple, high-efficiency, and sensitive electrochemical sensors have been developed for the detection of HbA1c. This review aims to highlight current methods and trends in electrochemistry for HbA1c monitoring. The target analytes of electrochemical HbA1c sensors are usually HbA1c or fructosyl valine/fructosyl valine histidine (FV/FVH, the hydrolyzed product of HbA1c). When HbA1c is the target analyte, a sensor works to selectively bind to specific HbA1c regions and then determines the concentration of HbA1c through the quantitative transformation of weak electrical signals such as current, potential, and impedance. When FV/FVH is the target analyte, a sensor is used to indirectly determine HbA1c by detecting FV/FVH when it is hydrolyzed by fructosyl amino acid oxidase (FAO), fructosyl peptide oxidase (FPOX), or a molecularly imprinted catalyst (MIC). Then, a current proportional to the concentration of HbA1c can be produced. In this paper, we review a variety of representative electrochemical HbA1c sensors developed in recent years and elaborate on their operational principles, performance, and promising future clinical applications.

## 1. Introduction

Diabetes mellitus (DM) is one of the three most harmful non-communicable diseases to humans [1], with an estimated global prevalence of 9.3% (463 million people) in 2019, which is projected to increase to 10.2% (578 million) by 2030 [2]. DM is a group of metabolic diseases associated with high blood glucose [3]. Conventionally, diabetes detection is based on glucose sensing, which is a continuous process, such as impaired fasting glucose (IFG) and impaired glucose tolerance (IGT), and which can easily cause diagnosis errors [4,5]. However, more recently, glycated hemoglobin (HbA1c) has been shown as an index of blood glucose levels in patients in the past 60 to 90 days, and therefore, it could be an excellent biomarker for continuous glucose monitoring. This protein is a stable product of a non-enzymatic reaction of glucose and human hemoglobin (Hb) β-chain N-terminal valine in serum, and its concentration is insensitive to short-term fluctuations in glucose [6,7,8]. Therefore, HbA1c levels reflect the long-term glucose levels of a patient, which can improve diabetes diagnostic accuracy [9] and is crucial for the diagnosis of diabetes [10]. HbA1c levels are defined as the ratio of HbA1c concentration to total hemoglobin concentration and are ~4–6.5% for a normal person, while the clinical reference range for its concentration is 5–20% [4], and the physiological levels range from 3 to 13 mg/mL in human blood samples [5]. In addition, current diagnostic criteria for diabetes include the requirement of monitoring fasting blood glucose or plasma glucose measured 2 h after an oral glucose tolerance test (OGTT). By contrast, HbA1c is more convenient, requiring no preparation, and has the lowest intra-individual variation [11].

Several clinical methods are currently available for determining the level of HbA1c in bodies, including liquid chromatography [12], electrophoresis [13], affinity chromatography [14], ion exchange chromatography [15], and immunoassays [16]. Although the effectiveness of these methods has been demonstrated in clinical practice, they require expensive and professional equipment, operation by experienced professionals, and complicated testing processes [17]. In contrast, electrochemical methods require no professional equipment or well-trained operators, and the testing processes are simple and quick [18]. Furthermore, using captured biomolecules, proteins, or antibodies to activate the surface of the electrodes and enable repeatable electrical output for HbA1c detection has significant applications in point-of-care testing (POCT) [19,20]. Here, we focus exclusively on electrochemical sensing and biosensing reports of HbA1c detection by using different materials.

**Figure 1:**
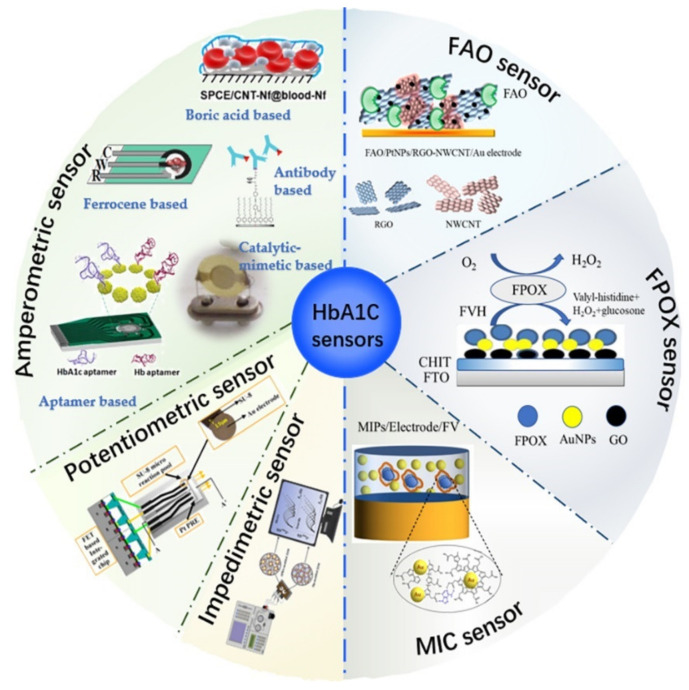
Classification of electrochemical sensors for HbA1c detection.

Depending on the target analyte, electrochemical-based HbA1c sensors can be divided into two categories: “direct sensors,” based on detecting HbA1c directly, and “indirect sensors,” based on detecting FV/FVH indirectly [7,21,22]. With the direct sensors, bio-affinity molecules are pre-modified on the surface of electrodes to capture HbA1c, and then, the concentration of HbA1c can be directly determined according to the change in electrical signals generated from electrochemical reactions [23]. Moreover, based on the detection signals, direct type sensors can be further classified into three main types: (a) amperometric sensors, (b) potentiometric sensors, and (c) impedimetric sensors [23]. On the other hand, indirect sensors are built to detect the FV/FVH released from HbA1c by protease digestion [24], which has a specific proportional relationship with HbA1c in terms of moles [25]. Indirect sensors are also based on the measurement of electrical signals generated by the redox reaction during the oxidation of FV/FVH by FAO/FPOX/MIC, which is directly proportional to the concentration of HbA1c [26]. To the best of our knowledge, existing electrochemical indirect sensors of HbA1c provide only electrical currents as output; therefore, we will classify these sensors based on the enzyme types. Specifically, three major enzyme types will be discussed: (1) FAO type, (2) FPOX, type and (3) MIC type. The overall classification of electrochemical HbA1c sensors discussed in this paper is shown in Figure 1.

According to the indicators of general household testing equipment, the performance and potential applications of HbA1c biosensors in diabetes diagnosis can be evaluated from five perspectives: (1) suitability of their detection range for clinical use, (2) detection limit, (3) detection time, (4) sensitivity, and (5) continuous stability [27,28].

In this paper, we intend to provide a comprehensive review of the advances in electrochemical sensors for HbA1c detection in recent years and compare the advantages and disadvantages of these sensors from the above five perspectives. Finally, we elaborate on the challenges to address in the development of commercially successful HbA1c sensors, such as sensitivity, stability, continuity, and in situ monitoring in a complex environment.

## 2. Direct Type Electrochemical HbA1c Sensors

Direct type sensors determine HbA1c by detecting the changes in electrical signals including current, potential, and impedance before and after HbA1c is bound to biological affinity molecules pre-fixed on the electrode surface. Direct sensors are divided into amperometric sensors, potentiometric sensors, and impedimetric sensors. 

### 2.1. Amperometric Sensors

The amperometric HbA1c sensor detects biomolecules by the change in current as the output signal. This type of sensor was first developed in 2002, in which HbA1c molecules were attached to the electrode surface by a cellulose membrane pre-modified with globin [29]. This ground-breaking work verified the capability and promising potential of amperometric sensors for detecting HbA1c. Moreover, antibodies, boric acid and its derivatives, ferrocene and its derivatives, and nucleic acid aptamers can be used to construct amperometric sensors [22,25,30]. Since HbA1c is a protein with reduction property, in general, the detection mechanism of the amperometric HbA1c sensor is that the electrode modification substance oxidizes HbA1c to produce a redox reaction, or HbA1c hinders the oxidation current value of redox media [31,32].

**Figure 2:**
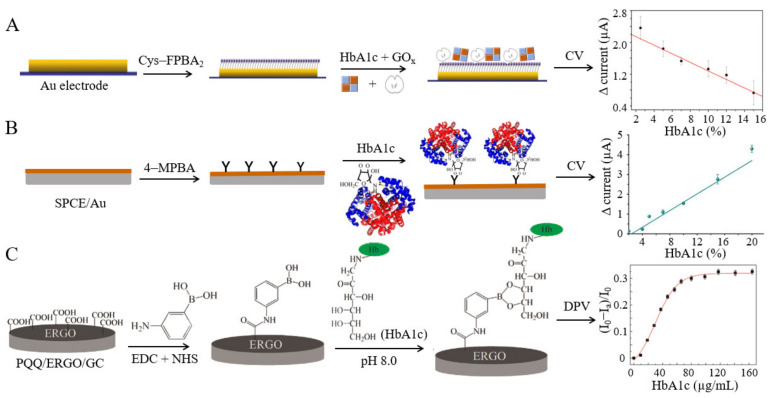
(**A**) Electrochemical sensor based on the HbA1c and GO_x_ competition mechanism, as well as the ΔCV responses of HbA1c (this figure was adapted from [35] with some modifications); (**B**) Electrochemical sensors based on 4-MPBA specific recognition and the ΔCV responses of HbA1c (this figure was adapted from [42] with some modifications); (**C**) Recognition of HbA1c of PBA-PQQ/ERGO/GC electrode and linear calibration plot of I_d_ value vs. the concentration of HbA1c (this figure was adapted from [40] with some modifications).

**Boric acid-based.** Under weakly alkaline conditions, boric acid can covalently bind to the diastatic cis-diol bonds on the surface of HbA1c [33]. Song et al. [34] proposed a method of using boric acid-polyamine G4 dendrimer-modified electrodes, verified successful binding of HbA1c at a content ranging from 2.5% to 15%, and simultaneously measured the electrochemical current generated as ferrocene methanol was catalyzed by glucose oxidase (GO_x_) on the electrode surface. The current value can be used as an indicator of the combination of HbA1c and the boric acid layer. Furthermore, to detect HbA1c in whole human blood, Song et al. developed a competitive electrochemical HbA1c biosensor based on the cis-diol interaction between HbA1c and a boronate recognition group [35]. Predetermined concentrations of HbA1c and activated GO_x_ were simultaneously dropped onto the surface of boric acid-modified electrodes, and the two species competed for the limited binding sites (Figure 2A). The experimental results provided a linear response within the content range of 4.5–15%. This biosensor holds great potential for the determination of HbA1c in whole blood samples without labeling with antibodies, dyes, or fluorescent materials. However, since boric acid can also combine with other sugar substances [36], the detection specificity is poor, and the entire blood sample needs to be pretreated before detection. Recently, a certain modification of boric acid was performed to achieve specific binding of HbA1c. Thiruppathi et al. developed a dual-electrode sensor (SPCE/CNT-Nf@Hb-Nf and SPCE*/AQBA-HbA1c) to detect Hb and HbA1c in whole blood samples simultaneously. Anthraquinone boric acid was prepared through electrooxidation with anthracene boric acid as the raw material. The SPCE*/AQBA electrode could be identified using a specific borate-diol and recombined with the applied HbA1c [18].

Phenylboronic acid (PBA) is obtained by replacing one of the hydroxyl groups in boric acid with a phenyl group. This compound can bind HbA1c by a borate bond, and the catalyst or redox-active species should be attached to the electrode to obtain an electrical signal [37]. A poly(terthiophene benzoic acid) (pTTBA)-modified electrode was immersed in the HbA1c solution, and the current generated from the reduction reaction between HbA1c and hydrogen peroxide (H_2_O_2_) was used to determine the HbA1c level. The linear dynamic detection range varied only from 0.1 to 1.5% [38], and it was not suitable for clinical use. Another research group, Chopra et al., used a conducting self-assembled monolayer (SAM) of mercaptophenyl boronic acid (MPBA) to bind HbA1c [39]. The authors labelled a gold screen-printed electrode with a ferrocene-tagged anti-HbA1c antibody (FcAb) as a tracer molecule. The produced current was proportional to the amount of HbA1c between 5% and 16%, significantly expanding the detection range. In another study, researchers coated a layer of phenylboronic acid-modified pyrroloquinoline quinine (PBA-PQQ) onto the surface of a glassy carbon disc electrode [40] (Figure 2C). The HbA1c captured on the electrode surface led to the reduction in the oxidation peak current of PQQ, because the protein molecules hinder the electron transfer pathway. Within the HbA1c concentration range of 9.4–65.8 µg/mL, the peak current decreased linearly by differential pulse voltammetry (DPV). Similarly, carbon electrodes coated with polyaniline boric acid nanoparticles were also used to determine HbA1c in a label-free manner [41]. The peak current varied according to a linear relationship with the logarithm of HbA1c concentration within the range of 0.975–156 µM, with high selectivity.

In recent years, some novel electrochemical HbA1c sensors have been reported. A reticulated vitreous carbon (RVC) electrode was modified with 3-aminophenylboronic acid, chitosan (CHIT), and tetraethyl silica (TEOS) [32]. The biosensor was employed to detect HbA1c in clinical samples, and comparison showed that the detection results were basically the same as those from automatic biochemical analysis. In 2018, a molecularly imprinted polymer (MIP) flexible sensor was reported to simultaneously detect HbA1c and Hb by specifically capturing targets through non-covalent bonding and a cis-diol structure [43]. The linear ranges for detecting HbA1c and Hb were 0.2–230 ng/mL and 0.5–200 ng/mL, respectively. The sensor was successfully applied to determine the concentration of HbA1c in blood samples collected from women with gestational diabetes and healthy pregnant women. A novel graphene-doped titanium dioxide (TiO_2_)-based heterojunction nano hybrid material (HJNH) was modified by poly(3-aminophenylboric acid) (PAPBA) and gold nanoparticles (AuNPs) [44]. The boric acid group in PAPBA was used to capture HbA1c. When the content of HbA1c ranged from 2.0% to 10%, the signal was directly proportional to the electrocatalytic reduction current of H_2_O_2_, and the detection limit was 0.17%. In 2019, another type of unlabeled electrochemical sensor was developed for HbA1c detection. In this sensor, 4-mercaptophenyl boric acid (4-MPBA)-modified screen-printed electrodes modified by gold nanoflowers (AuNFs) were used as the sensing electrodes [42] (Figure 2B). The linear range for detecting HbA1c was 5–1000 µg/mL or 2–20% of the content, and the results demonstrated that the sensor had good specificity and stability. In addition, the use of a 16-channel screen-printed electrode and the considerably reduced detection time and cost endowed this sensor with significant performance superiority over other existing HbA1c detection methods in terms of pretreatment and operation procedures. Recently, to improve the electrode surface electron transmission capacity, Li et al. designed a three-dimensional antifouling nano-biosensing surface based on bovine serum albumin (BSA) and glutaraldehyde (GA) cross-linking and then used the HbA1c antibody and 3-aminophenyl boronic acid (APBA) to functionally modify the surface [45]. The presence of non-glycated hemoglobin (HbAo) resulted in a linear dynamic range of 2–15%, which facilitated label-free POCT detection of HbA1c. A redox medium was fixed on the surface of a nanocomposite, which contained pTTBA and N,S-doped porous carbon (NSPC), to fabricate HbA1c sensors [46]. This system could accurately separate and detect Hb and HbA1c in blood samples. The linear dynamic ranges of Hb and HbA1c were 1.0 × 10^−6^–3.5 mM and 3.0 × 10^−6^–0.6 mM, respectively.

**Ferrocene-based.** Ferrocene (Fc)-modified electrodes may be promising for the construction of current sensors because of the reasonable stability and structural versatility of Fc derivatives [23,47,48]. In a recent study, Han et al. reported a novel scheme that made full use of the redox ability of ferrocene diformylcysteine (Fc[CO-Cys(Trt)-OMe]_2_) and ferrocene glutathione (Fc[CO-Glu-Cys-Gly-OH]) [49]. The two derivatives were adsorbed on the surface of electrodes modified by AuNPs, and the performance of the sensor was quantitatively characterized. The ferrocene glutathione sensor was proven to have a stronger catalytic current response to Hb, and the current showed a good linear correlation, with Hb concentrations ranging from 0.1 to 1000 µg/mL. The relative standard deviation was less than 4.7%, and the recovery rate was between 95.5% and 103.2%. Both properties meet the clinical requirements for Hb analysis.

**Aptamer-based.** A nucleic acid aptamer is a sequence of short single-strand DNA or RNA, and aptamers are produced through in vitro selection procedures. Aptamers provide several unique advantages: minimal possibility of chemical synthesis, small batch variability, long shelf life, stability under various conditions, and a variety of available chemical modifications. Therefore, aptamers are now the most promising alternative to monoclonal antibodies [50]. Novel aptamers for glycated and total hemoglobin have been selected recently, showing high affinity and specificity [51].

**Figure 3:**
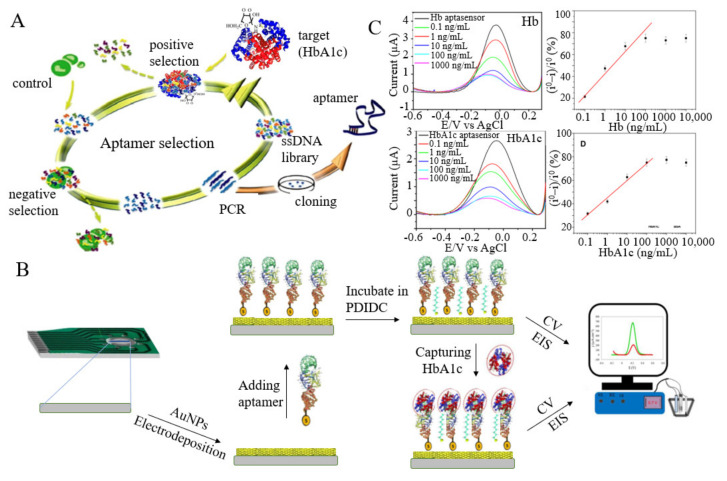
(**A**) SELEX was used to screen suitable molecules bound to HbA1c. (**B**) The manufacture and measurement of the HbA1c sensor (reprint permission has been requested from [52], and it was also adapted from [53]). (**C**) SWV diagram and calibration curve of suitable aptamers of Hb and HbA1c junctions at different concentrations (reprint permission has been requested from [52]).

Kim et al. reported a dual sensor for detecting HbA1c and Hb in a finger prick blood sample (1 µL) [17]. The Hb content was determined by measuring the cathode current generated from catalysis with toluidine blue O (TBO), while the HbA1c content was determined by measuring the cathode current produced when HbA1c was captured by the aptamer. The dynamic ranges for detecting Hb and HbA1c were 0.1–10 µM and 0.006–0.74 µM, respectively, and the mean HbA1c values (%) of the proposed method were also proven to be reasonable by comparison with high performance liquid chromatography (HPLC). Shimaa et al. screened two specific aptamers with dissociation constants of 2.8 nM and 2.7 nM for HbA1c and Hb, respectively, based on the systematic evolution of ligands by exponential enrichment (SELEX) process [52]. Then, the authors fixed the sulfhydryl-modified aptamers onto the surface of array electrodes modified by AuNPs to perform label-free detection of HbA1c and Hb (Figure 3A–C). The sensor had a high sensitivity and detected HbA1c and Hb with detection limits of 0.2 ng/mL and 0.34 ng/mL, respectively. Such an array platform is superior to the existing immunoassay methods due to its simplicity, stability, low sample consumption, and low cost. However, the main disadvantage is that has a complicated operation. This method can detect HbA1c in human whole blood without any pretreatment and has broad applications in the diagnosis of diabetes.

Aptamer-type sensors can detect HbA1c specifically, but they usually require complex electrode modification. Shajaripour et al. proposed an electrochemical nano-genosensor, in which a reduced graphene oxide (RGO)-gold nanostructure was facilely electrodeposited on a graphite sheet (GS) electrode, and then, vulcanized DNA aptamers were fixed on the electrode surface [53]. The sensor had a high sensitivity of 269.2 µA/cm^2^, a wide linear range of 1 nM–13.83 µM, and a low detection limit of 1 nM. This sensor has been successfully applied in blood samples and is expected to be a promising tool for diabetes screening and management. Furthermore, due to a large amount of carbohydrates and protein in whole blood samples, the effective antifouling ability of the electrode can improve the affinity and specificity of detection. Duanghathaipornsuk et al. [54] developed a gHb-targeted aptamer (GHA) through a modified SELEX process, and it was used to produce three distinct SAM-SPR-sensing surfaces with and without an antifouling layer. The results showed that the correlation between the HbA1c-targeted aptamer and HbA1c of the sensor surface with antifouling modification was higher than that of the sensor surface without modification, and the interference of nonspecific protein adsorption was reduced. This system illustrates the role of aptamers and antifouling surface modifications in developing effective, low-cost, and rapid HbA1c analyses in blood samples.

**Antibody-based.** Specificity and simplicity are the greatest advantages of this kind of immunosensor, which can satisfy the detection of HbA1c in a complex sample environment. Liu et al. fabricated a mixed layer on glassy carbon, which was attached by the redox probe 1,1′-di(aminomethyl)ferrocene (FDMA), followed by covalent attachment of the epitope glycated pentapeptide (GPP), an analogue to HbA1c, to promote competitive inhibition between antibodies and HbA1c [55]. A good linear relationship was observed between the relative faradaic current of FDMA and the concentration of HbA1c, ranging from 4.5% to 15.1% of the total hemoglobin in the serum, without the need for washing or rinsing steps. In addition, the preparation technology of antibodies is complex, time-consuming, and expensive. Karaşallı et al. [56] used a reduced graphene oxide (ERGO)-modified glassy carbon electrode (GCE) as a sensing interface and dropped an anti-HbA1c antibody solution onto the GC/ERGO electrode. A linear relationship was obtained between the DPV response and HbA1c concentrations from 1% to 25%. Alireza et al. [57] used a 3-mercaptopropionic acid (MPA) self-assembled monomolecular membrane to covalently attach anti-HbA1c antibodies. This binding process occurred on the gold electrode surface, which was previously coated with a polyethylene terephthalate (PET) substrate. For samples of HbA1c dissolved in 0.1 M PBS, this sensor had a dynamic range of 7.5–20 µg/mL. For undiluted human serum samples, a linear correlation was observed in the range of 0.1–0.25 mg/mL HbA1c. These results demonstrated that the sensor holds great potential in the treatment of diabetes in the future.

**Catalytic-mimetic-based.** In addition to boronic acid and its derivatives, ferrocene and its derivatives, nucleic acid aptamers, and antibodies, there are some other types of materials suitable as sensitive materials for fabricating electrochemical HbA1c sensors [58]. For example, flexible conductive artificial enzymes can be used for HbA1c detection. Conductive artificial enzyme nanoparticles were prepared by molecular imprinting technology [59]; in the presence of Rhodamine b and 3-aminophenyl boronic acid, Hb and HbA1c were embedded into the molecularly imprinted polymer, and then they were removed to form specific 3D binding sites in the polymer. The catalytic-mimetic HbA1c biosensor based on the lock-key model has good specificity and promotes the redox process. The linear ranges for HbA1c and Hb detection were 0.5–100 mM and 0.45–120 mM, respectively, and only 0.07 µL of the sample was required for one test.

### 2.2. Potentiometric Sensors

Potentiometric HbA1c biosensors mostly include immune sensors based on integrated chips or extended gate electrode arrays as sensitive elements [60], and they can simultaneously detect Hb and HbA1c without markers. The integrated chip is built based on standard complementary metal oxide semiconductor (CMOS) technology. Before detection, anti-HbA1c antibodies are coupled with ion field effect transistors [61]. The detection mechanism of the potentiometric HbA1c sensor is that on the gate of the field effect transistor, the biofilm is deposited to form a double electric layer, and its potential changes along with the concentration of HbA1c [62,63].

**Figure 4:**
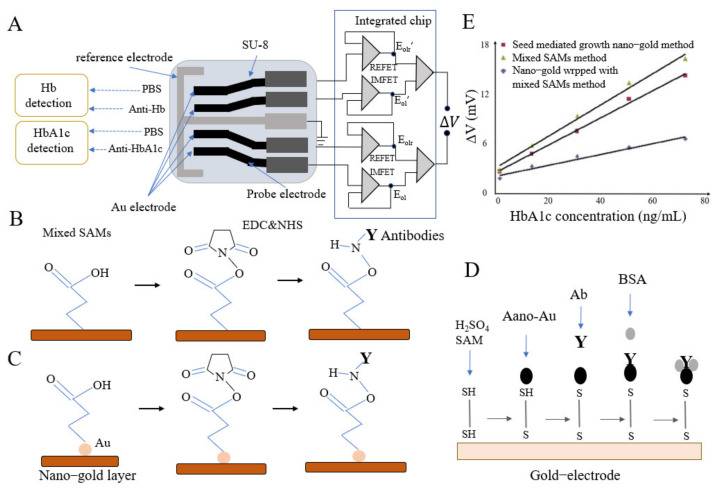
(**A**) Schematic diagram of HbA1c microsensor; (**B**) Mixed SAMs method; (**C**) Seed mediated growth nano-gold method; (**D**) HbA1c test strip prepared by SAM and nanotechnology; (**E**) Voltage responses of three kinds of immunosensor in the simulated blood sample to HbA1c (reprint permission of A~E has been requested from [63,64]).

Xue et al. built a potentiometric label-free immune microsensor using the CMOS process [63]. This sensor was composed of a CMOS with a micro-signal readout circuit and disposable test strip electrodes (Figure 4A). Using a self-assembled monolayer film coated with AuNPs, the authors fixed antibodies onto the electrode surface (Figure 4B,D) and detected HbA1c and Hb within linear ranges of 4–24 mg/L and 60–180 mg/L, respectively. Furthermore, an improved AuNPs sensor with SAMs eliminated nonspecific sites and interference (Figure 4B–E). Compared to an immunosensor fabricated by the mixed SAMs method and without gold nanofilm, this sensor had twofold higher sensitivity [64]. Another group built a disposable potentiometric immune sensor using screen-printed electrodes and PET [65]. To improve the sensitivity by exposing HbA1c to the antibodies, 0.2% ammonium dodecyl trimethyl bromide was added to denature HbA1c. This sensor was successfully applied in the direct determination of HbA1c, and the results showed a good correlation between the HbA1c standard and measured values.

Additionally, alizarin red s (ARS) can be used as an indicator of the redox reaction in the potential detection of HbA1c [66]. A negative redox potential shift was produced by binding PBA to ARS and HbA1c after a complex reaction with dialcohol-boric acid [67]. First, the potential of the ARS-PBA complex was negative. After competition between HbA1c and ARS to bind PBA, however, the potential shifted positively, and the shift value was related to the HbA1c concentration. The concentration of HbA1c measured according to the potential changes was in good agreement with the reference results.

### 2.3. Impedimetric Sensors

Accurate and rapid detection has always been a research hotspot in the field of medical diagnosis. Impedance sensors are effective in detecting the reaction mechanism of the modified electrode interface and provide a fast detection method by studying the conductivity and chemical conversion process with electrochemistry [68]. In general, the detection mechanism of impedimetric HbA1c sensors is that the accumulation of HbA1c on the biosensor film changes the resistance characteristics of the electrode interface [68]. These sensors can detect affinity interactions (e.g., antibody-antigen interactions) without labeling in real time [69,70,71,72]. Park et al. reported a novel sensor with HbA1c immobilized on a gold electrode covered by an SAM of thiophene-3-boronic acid (T3BA), using K_3_Fe(CN)_6_ and K_4_Fe(CN)_6_ as a redox probe. The rate of charge transfer between the electrode and the redox probe is related to the concentration of HbA1c [73]. Since HbA1c is difficult to distribute evenly on the sensor surface, the stability of the detection results is not excellent. Although the redox agent can significantly improve electron transfer, it may also diminish the activity of the electrode/SAM interface over time.

Fortunately, Hu et al. found that the electrode could detect surface binding behavior within a specific frequency range, even without a special redox reagent [74]. Chuang et al. [75] achieved the detection of HbA1c without redox reagent in the frequency range of 20–1000 Hz. The sensor consisted of a pair of parallel electrodes integrated into a microfluidic device that was modified by an SAM of T3BA. This sensor can be easily integrated into a microfluidic device, consuming a low amount of the sample. Furthermore, Hsieh et al. [76] proposed a circular gold finger-like electrode on this basis, which still required no redox reagents. The electrode could measure HbA1c concentrations from 1 to 100 ng/µL at frequencies ranging from 0.5 to 20 kHz. This strategy makes it more suitable for POCT applications. Moreover, Boonyasit et al. proposed a novel 3D paper-based electrochemical impedance device combined with haptoglobin (Hp)-modified and APBA-modified eggshell membranes (ESMs) that was highly responsive within the clinically relevant total concentration range (0.5–20 g/dL) and that of HbA1c (2.3–14%) and reduced the data acquisition time 15-fold [77]. This micro-fast sensor not only shows great potential for POCT but is also a unique platform for off-site clinical diagnosis. The interface materials, detection mechanisms, and detection results of direct type sensors were compiled and are shown in Table 1. 

## 3. Indirect Type Electrochemical HbA1c Sensors

Indirect type electrochemical HbA1c sensors work based on the measurement of FV or FVH, which is a form of enzymatic determination. According to the type of enzymes, indirect sensors are divided into FAO type, FPOX type, and MIC type sensors. These enzymes usually need to be processed with nanotechnology or imprinting technology to immobilize them on the electrode surface. The enzymatic determination of HbA1c entails the following three steps [78,79,80,81,82]:

(1) Enzymatic hydrolysis: HbA1c is proteolytically decomposed, and its β-chain glycated nitrogen terminus is hydrolyzed to produce FV or FVH.
(2) Enzymatic catalysis: FV and FVH are oxidized to produce H_2_O_2_ by FAO and FPOX. In addition, in indirect MIC type sensors, FV usually participates in a redox reaction with other electronic mediators and does not produce H_2_O_2_.
(3) H_2_O_2_ determination: The produced H_2_O_2_ can be determined electrochemically, and the results are further used to determine the content of HbA1c in samples. The process can be described by steps (1)–(4), as follows.
  (1) $$\mathrm{HbA}1c\overset{\mathrm{protease}}{\to }\mathrm{FV}\;\mathrm{or}\;\mathrm{FVH}+\mathrm{amino}\;\mathrm{acid}\;$$
  (2) $$\mathrm{FV}+O_{2}+H_{2}O\overset{\mathrm{FAO}}{\to }H_{2}O_{2}+\mathrm{Valine}+D-\mathrm{Glucosone}\;$$
  (3) $$\mathrm{FVH}+O_{2}+H_{2}O\overset{\mathrm{FPOX}}{\to }H_{2}O_{2}+\mathrm{Valine}\;\mathrm{histidine}+D-\mathrm{Glucosone}$$
  (4) $$H_{2}O_{2}\to 2H^{+}+O_{2}+H_{2}O\;$$

### 3.1. FAO Type

Currently, FAO has been proven to be reproducible [83,84,85] and suitable for simple, convenient, and economical real-time HbA1c detection [85]. The emergence of nanotechnology has led to a new generation of electrochemical biosensors with nanostructured interfaces that enable faster detection with smaller volumes [86]. Currently, a wide range of nanomaterials, such as gold, silver, carbon nanotubes, graphene, and metal oxides, have been successfully applied in biosensors [87]. These materials have excellent properties, such as high biocompatibility, good water dispersibility, and large surface areas [88,89,90]. Doping nanoparticles into normal materials or substrates can improve the resulting electrochemical properties [91].

**Figure 5:**
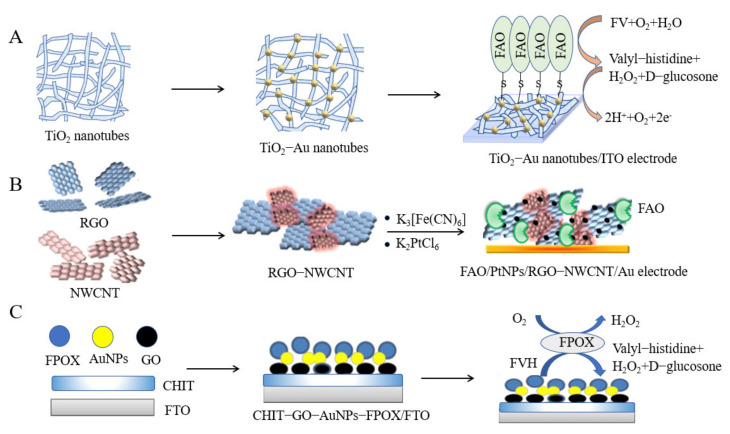
(**A**) An FAO/AuNP-PTA-TiO_2_ nanocomposite was prepared on an ITO electrode (this figure was adapted from [92], with some modifications); (**B**) Preparation method of the FAO/PtNPs/RGO-NWCNT nanocomposite (this figure was adapted from [94], with some modifications); (**C**) CHIT-GO-AuNPs-FPOX nanocomposites were prepared on an FTO glass plate (this figure was adapted from [104], with some modifications).

Utkars et al. [92] formed a stable scaffold with AuNPs and point-like tubular TiO_2_; 12-phosphotungstic acid was used as a reductant after depositing well-dispersed AuNPs on TiO_2_ nanotubes, which accelerated electron transfer between proteins and conductors (Figure 5A). The response time was only three seconds, and the linear range for HbA1c detection was 0.5–2000 µM. Furthermore, Utkarsh et al. [93] fabricated a fixed nitrogen-doped graphene/AuNP/fluorine-doped tin oxide (FTO) glass electrode based on fructosyl amino acid oxidase (FAO), with a half-life of up to four months. This biosensor could detect HbA1c in human whole blood, exhibiting a low detection limit of 0.2 μM.

To further improve the reaction efficiency, Utkarsk et al. [94] synthesized 3D-structured reduced graphene oxide, multi-walled carbon nanotubes, and platinum nanoparticles (PtNPs) (Figure 5B) to coat an Au electrode. Additional reaction sites were exposed, the response time of the sensor was reduced to less than three seconds, and the linear range was 0.05–1000 µM. This sensor had good repeatability and was successfully used to determine the concentration of HbA1c in human blood samples. Moreover, Utkarsh et al. mixed AuNPs-PtNPs and polyindole-5-carboxylic acid (PIN5COOH) and modified them onto the surface of a gold electrode [95], which showed good storage stability and retained 50% of the initial activity after 12 weeks. The unique characteristics of the two different metal nanoparticles helped improve the sensitivity and specificity of the sensor; its linear detection range for FV was 0.1–1000 µM, and its detection limit was 0.2 µM. Sheetal et al. prepared a film of a ZnO nanoparticle/polypyrrole hybrid [96] and fixed it onto a gold electrode surface with FAO. The sensor had a low detection limit of 0.05 mM, and its linear detection range for FV was 0.1–3 mM. The modified electrodes could be stored for 160 days and be used to analyze human whole blood samples.

Magnetic nanoparticles have drawn considerable research attention as special biomolecular immobilized carriers [97,98]. Sheetal et al. introduced amino groups onto the surface of core-shell magnetic nanoparticles [99]. The sensor was built by immobilizing FAO on the modified nanoparticle surface to achieve high sensitivity. The linear detection range for FV was 0–2 mM, and the detection limit was as low as 0.1 mM. Moreover, this enzyme electrode could be stored for three months and used up to 250 times.

Recently, some semiconductor nanomaterials have been applied in the research of nanometer sensors due to their good stability [100]. Chauhan et al. [101] established a direct, rapid, and sensitive HbA1c sensor, which was constructed by immobilizing FAO onto a ZnO nanorod-modified indium tin oxide (ITO)-coated glass plate electrode. The sensor presented significant sensitivity and detection limit advantages (0.1 µM), a fast response time (4 s), and a wide linear range (0.1–2000 µM). The working electrode was stable for approximately 4 months at 4 °C. This sensor could be used to distinguish HbA1c in blood samples from healthy and diabetic patients.

In addition, Leng et al. fabricated an amperometric biosensor by drop-coating an FAO enzyme onto an SPE surface [102]. The biosensor showed high current output, high linearity, and effectiveness for FV (0–8000 µM), as well as human blood samples. The drawback of the Prussian blue (PB) electrode for indirect HbA1c sensors is that Fe^3+^ in PB easily reacts with OH^−^ in solution [103]. Shi et al. utilized tris(hydroxymethyl) aminomethane to modify PB on the SPE surface [103] because OH and NH_2_ functional groups in tris can be complexed with Fe^3+^ in PB to avoid reactions of Fe^3+^ and OH^−^ in solution. The modified Tris-PB/SPE was applied in the detection of H_2_O_2_, presenting a linear range of 0–2000 µM FV.

### 3.2. FPOX Type

Compared with other enzymatic HbA1c sensors, FPOX type HbA1c sensors have high specificity because substantial specific measurements of HbA1c can be realized through mutagenesis and modification [104,105]. As shown in Figure 5C, Shahbazmohammadi et al. immobilized a recombinant engineered FPOX enzyme to specifically hydrolyze FVH on an electrode surface modified by CHIT, graphene oxide (GO), and gold nanoparticles (AuNPs). The biosensor showed a linear response within the range of 0.1–2 mM [104]. In addition, to prove that the FPOX-modified electrode can specifically measure FVH, significant changes in electron transfer resistance were observed after incubation of the FPOX-modified electrode with FVH, but there was no response in the control group, indicating the specific measurement of FVH. Hatada et al. replaced Arg414 with Lys to form the PnFPOX (FPOX from Phaeosphaeria nodorum) N56A/R414K mutant and modified PnFPOX near FAD with amine-reactive phenazine ethosulfate (arPES), which showed quasi-direct electron transferability [106]. This electrode was combined with an enzyme flow injection analysis (FIA) system. The linear range of the system for both FV and FVH was 20–500 μM, and the sensitivities and detection limits of the system were 0.49 nA·μM^−1^ and 1.3 μM for FV, respectively, and 0.13 nA μM^−1^ and 2.0 μM for FVH. The oxidative activity and specificity of PnFPOX for this method are commendable, but the detection range needs to be expanded if this method is to be adopted for clinical measurements of HbA1c. In addition, the PnFPOX electrode was engineered for continuous operation of the FIA system. This case shows that an electrochemical sensor can be combined with an FIA system to develop an integrated measurement system for sample pretreatment and sample electrochemical measurement.

There are also some sensors that modify both FPOX and FAO, which can selectively determine only glycated N-terminal peptides from the β chain without any interference in the presence of glycated peptides from the α chain [107]. Nanjo et al. adopted an FIA system with a flow-type spectrophotometer and electrochemical detector (with FPOX immobilized on amino-alkyl-bonded Uniport C and FAO immobilized on dialdehyde-activated Uniport C) [107]. The total hemoglobin in the sample is determined by spectrophotometry, and then, the FVH released from HbA1c is determined by an electrochemical sensor with FPOX or FAO. As a result, at pH 8.0–8.5, FAO showed high activity against FV but no activity against FVH, and at nearly pH 7.0, FPOX showed the maximum activity of FV and FVH. The FIA system with the FAO reactor showed a linear detection range for FV of 2–200 µM, and the FPOX reactor showed linear detection ranges for FV and FVH of 2–100 µM and 7–110 µM, respectively. However, when blood cell samples were digested by protease, only FVH was released, not FV. Therefore, according to the definition of HbA1c by the International Federation of Clinical Chemistry (IFCC), the combination of protease and FPOX reactor systems can determine only FVH.

### 3.3. MIC Type

**Figure 6:**
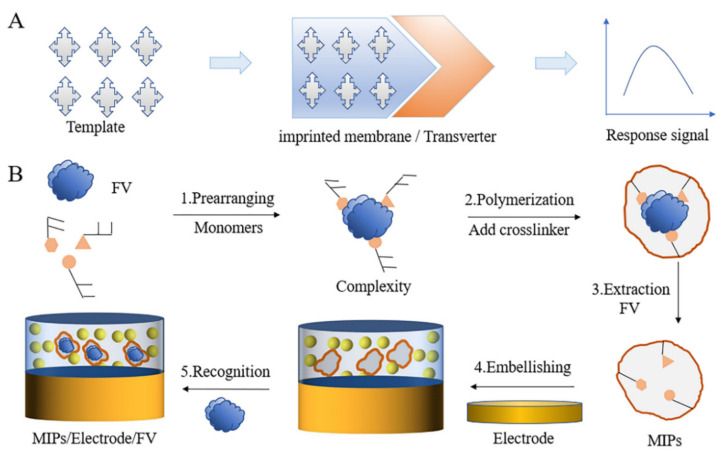
(**A**) The working principle of the molecularly imprinted sensor; (**B**) A molecularly imprinted sensor that specifically recognizes FV (this figure was adopted from [108]).

The molecular imprinting catalyst is the formation of molecular recognition sites in a polymer by performing synthesis in the presence of a target template. Therefore, MIPs can selectively identify target analytes. The use of MIP technology is the most common, stable, scalable, and economical method at present [108,109]. In this technology, a functional monomer is combined with imprinted molecules and fixed by a cross-linking agent, and then, the imprinted molecules are washed away. The goal is to develop a polymer material with high affinity and selectivity to bind the target substance (Figure 6) [82,108]. The content of the template monomer used in the preparation process is usually less than 5%, while that of the cross-linking agent is as high as 95% [110].

MIC is an artificial enzyme catalyst. Molecular imprinting technology can be used to construct molecular recognition elements, and the premise of detecting FV by sensor elements based on the enzyme method is to develop a catalytic center, which is used to mimic fructosamine dehydrogenase [81]. Koji et al. found that polyvinyl imidazole (PVI) could be used as an oxidant in the oxidation reaction of FV in the presence of electron receptors under alkaline conditions. Similarly, the same team used a carbon paste electrode to fix PVI and built an amperometric sensor to detect FV, with a linear response range of 0.02–0.7 mM [81]. These results indicated that PVI can be used as a catalyst for oxidizing fructosamine compounds and HbA1c. This team also prepared an artificial fructosyl amine dehydrogenase, a polymer catalyst prepared by copolymerizing allylamine, 1-vinylimidazole, and 4-vinylphenylboronate to improve the selectivity of MIC to FV, showing a typical response curve of amperometric enzyme sensors for FV in the range of 0.2–0.8 mM [111]. Subsequently, the team used a higher concentration of buffer solution to improve the selectivity and sensitivity of MIC to FV by changing the operating conditions [112]. When 100 mM of potassium phosphate buffer were used instead of 10 mM, the sensitivity ratio of FV and Fru-ε-Lys with the sensor increased significantly from 1.9 to 5.7. However, the synthesized MIC was characterized by low flexibility, and most of the active catalyst sites were located inside the polymer. Therefore, the FV oxidation activity of the MIC needs to be improved [113]. Tomohiko proved that changing the flexibility of the polymer chain can improve the FV oxidation activity of the MIC [113]. A more water-soluble polyacrylamide gel with a relatively low cross-linking degree and macroporous structure was used to improve the conformational flexibility of the polymer. This material was fixed on a gold electrode, and an amperometric sensor was prepared that could detect 0.05 to 0.6 mM FV. Therefore, this soluble MIC sensor achieves the detection range required for HbA1c measurement.

In brief, existing indirect type HbA1c detection sensors that work by detecting FV/FVH have several performance advantages. Specifically, the detection time is generally only 2–120 s; the pH value of the measurement system is near that of human physiology (7.0–7.5). The linear detection range of FV by indirect sensors based on nanotechnology can reach 3000 µM at most, and the detection limit can reach at least 0.05 µM. The linear detection range of FV by indirect sensors based on imprinting technology can reach 800 µM, and the minimum detection limit is 50 µM. Due to the unique properties of nanomaterials, including adjustable chemical properties, large surface-to-volume area, strong biocompatibility, good stability, excellent conductivity, high sensitivity, and non-participation in redox reaction, electrochemical HbA1c sensors based on nanotechnology have attracted extensive attention (e.g., see [87]). For the FAO- and FPOX-based sensors, graphene, AuNPs, core-shell magnetic nanoparticles, metal oxide nanolayers, and nanotubes are the common materials used as electrodes. Using novel nanomaterials as sensing and conduction materials, electrochemical sensors were developed and are expected to provide more convenient, accurate and reliable platforms for the diagnosis of diabetes in the future [88,89,90,114,115,116]. In the MIC type indirect sensors based on imprinting technology, the specific binding sites of the target molecules can be easily customized in the polymer network. At present, these sensors can cover the scope of clinical detection, but most of them are highly cross-linked rigid polymers with low flexibility, so their activity is far lower than that of natural enzymes. Molecularly imprinted microgels and other flexible sensors combined with artificial enzymes may promote the application of imprinted sensors in the clinic. In addition, electrochemical measurements can be combined with the FIA system, which has broad prospects in the field of HbA1c detection. The interface materials and detection results of direct type sensors are compiled in Table 2.

## 4. Comparison of the Characteristics of Direct and Indirect Electrochemical HbA1c Sensors

In direct type sensors, HbA1c binds to the electrode surface through the bio-affinity of antibodies, boric acid, etc., and then, the appropriate signal transduction mode is used to directly detect HbA1c [23]. The selective binding of HbA1c to the electrode surface produces electrochemical signals in terms of current, voltammetry, impedance, or potential patterns. Such fabricated sensors have a wide detection range, but the specificity is poor. Generally, whole blood samples need to be pretreated to obtain HbA1c for further determination. These sensors are suitable for long-term HbA1c level monitoring.

Indirect type HbA1c sensors work based on the glycated amino acid FV or FVH produced through the hydrolysis of HbA1c protein as detected by FAO, FPOX, or MIC. HbA1c must be predigested by proteolytic enzymes to produce FV fragments. Therefore, these sensors involve much more complicated pretreatment and post-detection conversion processes than their direct type counterparts. The FV sensors mainly modified by nanomaterials offer a wide detection range in line with clinical requirements and good stability, and they generally do not suffer from specificity problems. However, the distribution of nanoparticles on the modified interface is usually not uniform, which results in poor repeatability. Moreover, FV sensors built based on imprinting technology usually have a relatively narrow detection range. Due to the selectivity of specific polymers to the template FV molecules, this type of sensor has good specificity and repeatability, as well as a longer stability period. The fabricated FVH sensors combined with the FIA system offer a wide detection range in line with clinical requirements, good stability, and multi-sample continuous testing. In addition, the advantages and disadvantages of different types of electrochemical HbA1c sensors were compiled and are shown in Table 3.

## 5. Conclusions and Future Prospects

This review focuses on the different types of electrochemical HbA1c sensors developed in recent years, considering the working principles, construction methods, response results, clinical applications, strengths, and weaknesses. Regarding the detection range, the electrochemical HbA1c sensor can meet the detection demand of the percentage content of HbA1c, but it cannot directly measure the concentration in blood samples or serum, and samples need to be diluted or pretreated. From the detection target point of view, electrochemical HbA1c sensors can be divided into direct and indirect types. Direct type sensors use boric acid and its derivatives, ferrocene, antibodies, and other substances to detect HbA1c directly. Indirect methods detect the hydrolyzed product of HbA1c based on immobilized enzymes, including FAO, FPOX, and MIC. Advances in electrochemical technology have considerably reduced the complexity and cost of HbA1c detection. Electrochemical HbA1c sensors have a wide linear detection range and good repeatability and are suitable for the detection of HbA1c in clinical practice. Compared with commonly used glucose sensors, these sensors are more suitable for the long-term monitoring of blood glucose levels in diabetic patients.

Electrochemical sensors have become an attractive alternative to conventional biosensing systems in the development of hospital or home diagnostic devices. The dynamic detection range of current HbA1c sensors is 4–20%, covering the range of human HbA1c levels. As clinical medicine techniques continue to be developed, there will be increased demand for diagnosis accuracy and efficiency. To better meet the needs for diabetes diagnosis, we believe that the repeatability and stability of electrochemical HbA1c sensors need to be improved to satisfy market demand for the future development of these sensors considering detection accuracy. This consideration is a major obstacle to translating such sensors into clinical applications and bringing them to market.

Electrochemical HbA1c sensors have found broad applications in the medical field. With the advancement of artificial intelligence (AI) technology, we suggest that electrochemical sensors should be combined with AI to further facilitate the prevention of diabetes and daily physical examination. To date, many different machine learning techniques have been developed to predict and diagnose diabetes, such as support vector machines, random forests, logistic regression, and k-nearest neighbors. Cyclic voltammetry, alternating impedance, and square wave voltammetry can be used to obtain a large quantity of detection data. These data can then be preprocessed to extract features and build models for the accurate prediction of HbA1c and FV levels. AI can help improve the detection efficiency of electrochemical HbA1c sensors and reduce the manufacturing costs. A further goal should be to achieve immediate detection, the ability to analyze multiple components, and comprehensive health management for diabetic patients.

## Figures and Tables

**Table 1:** Direct type electrochemical HbA1c sensors.

Type	Electrode/Interface Material	Detection Range/Limit	Mechanisms of Detection	Sample	References
Amperomeric sensors	Dend-FPBA electrode/poly(amidoamine) G4 dendrimer, GO_x_	2.5–15%/NA	GO_x_ catalyzes the oxidation of ferrocenemethanol	HbA1c reagent	[34]
	Au/Si electrode/Cys-FPBA2, GO_x_	4.5–15%/NA	GO_x_ catalyzes the oxidation of ferrocenemethanol	Human whole blood	[35]
	GCE/ERGO, PBA-PQQ	9.4–65.8 µg/mL/1.25 µg/mL	HbA1c hinders the oxidation current of PQQ	Human whole blood	[40]
	SPE/3-aminophenylboronic acid, chitosan, tetraethyl, orthosilicate	20–2200 µg/mL/NA	HbA1c is oxidized	Human whole blood	[32]
	Gold SPCE/mercaptophenyl boronic acid, anti-HbA1c (Fc labeled)	5–16%/NA	MPBA-HbA1c captures anti-HbA1c (Fc labeled), Fc is oxidized	HbA1c reagent	[39]
	MIP nanocube-modified CP@Al foil/APBA, polyrhodamine b	0.2–230 ng/mL/0.09 ng/mL	MIP catalyzes the oxidation of HbA1c	HbA1c reagent	[43]
	16-channel SPCE/AuNFs, 4-MPBA	2–20%/5–1000 µg/mL/0.65%, NA	H_2_O_2_ catalyzes the oxidation of HbA1c	Human serum	[42]
	Array SPCE/AuNPs, thiol-modified aptamer	6.67–10.47%/NA	HbA1c hinders the oxidation current of [Fe(CN)_6_]^4−/3−^	Human whole blood	[52]
	GS/RGO-AuNPs, aptamer, MU	1 nM–13.83 µM/ 1 nM	HbA1c hinders the oxidation current of [Fe(CN)_6_]^4−/3−^	Human whole blood	[53]
	Au electrode/3-mercaptopropionic acid, anti-HbA1c	7.5–20 µg/mL/100–250 µg/mL/7.5 µg/mL, NA	HbA1c hinders the oxidation current of [Fe(CN)_6_]^4−/3−^	0.1 mM PBS/human serum	[57]
	MIP nanocube-modified CP@Al foil/human-made enzyme	0.5–100 mM/ 0.09 µM	Artificial enzyme catalyzes the oxidation of HbA1c	Human whole blood	[59]
Potentioetric sensors	Probe electrode/thioalcohol, AuNPs, anti-HbA1c	4–24 µg/mL/NA	Potential difference in sensing chip	HbA1c reagent	[63]
	Au electrode/mixed SAMs, EDC&NHS, anti-HbA1c	1.67–72.14 ng/mL/NA	Potential difference in sensing chip	Simulated blood sample	[64]
Impedimetric sensors	Interdigital electrode/thiophene-3-boronic acid	10–100 µg/mL/ 1 µg/mL	HbA1c affects impedance changes	HbA1c reagent	[76]
	Dual SPCE and magnetic paper/haptoglobin, APBA	2.3–14%/0.21%	HbA1c affects impedance changes	Human whole blood	[77]

**Table 2:** Indirect type electrochemical HbA1c sensors.

Type	Electrode Type/Interface Material	Detection Range (FV)	Detection Limit	Potential	Sample	References
FAO	ITO electrode/AuNP-PTA-TiO_2_ nanocomposites	0.5–2000 µM	0.5 µM	~0.06 V	Human whole blood	[92]
	FTO glass electrode/nitrogen-doped graphene, AuNPs	0.3–2000 µM	0.2 µM	0.2 V	Human whole blood	[93]
	Au electrode/PtNPs-RGO-NWCNT	0.05–1000 µM	0.1 µM	~0.1 V	Human whole blood	[94]
	Au electrode/AuNPs-PtNPs, poly-indole-5-carboxylic acid	0.1–1000 µM	0.1 µM	0.2 V	Human whole blood	[95]
	Au electrode/ZnONPs-polypyrrole	100–3000 µM	50 µM	0.27 V	Human whole blood	[96]
	Au electrode/amino, core-shell magnetic bionanoparticles	0–2000 µM	100 µM	0.05 V	Human serum	[99]
	ITO electrode/ZnO, N-5-azido-2-nitro-benzoyloxysuccinimide	0.1–2000 µM	0.1 µM	0.2 V	Human whole blood	[101]
	SPE/-	0–8000 µM	-	-	FV reagent	[102]
	SPE/tris(hydroxymethyl)aminomethane, Prussian blue	100–2000 µM	100 µM	-	HbA1c reagent	[103]
FPOX	FTO glass electrode/AuNPs, GO, CHIT	100–2000 µM(FVH)	0.3 µM	0.3 V	Human whole blood	[104]
	FIA/spectrophotometer, FPOX-CET detector	2.66–11.84% (HbA1c)	-	-	Human whole blood	[107]
	FIA, Au electrode/PES-modified engineered FPOX	20–500 µM (FV) 20–500 µM (FVH)	1.3 µM/2.0 µM	0 V	HbA1c reagent	[106]
MIC	Carbon paste electrode/polyvinylimidazole (PVI)	20–700 µM	20 µM	-	FV reagent	[81]
	GCE/molecularly imprinted catalyst	200–800 µM	-	-	FV reagent	[111]
	Au electrode/1-ethyl-3-(3-dimethylaminopropyl)-carbodiimide	50–600 µM	-	-	FV reagent	[113]

**Table 3:** Comparison of sensors performance.

Type	Advantages	Disadvantages	References
Boric acid amperometric sensor	Easy to be chemically modified Short response time Wide detection range Simple fabrication process	Poor specificity Needs pretreatment of blood samples	[32,34,35,36,37,38,39,40,41,42,43,44,45]
Ferrocene amperometric sensor	Good specificity Good selectivity Wide detection range High sensitivity	Iron ion is easy to oxidize and has poor stability HbA1c should be labeled	[23,47,48,49]
Aptamer amperometric sensor	Good specificity Easy to chemically modify Good stability	Complex manufacturing process Needs screen for aptamers High price	[50,51,52,53,54]
Antibody amperometric sensor	Good stability Good specificity Easy to purchase Good stability	Poor sensitivity Long manufacturing time High price Susceptible to temperature, pH	[55,56,57]
Potentiometric sensor	High sensitivity Good stability Short response time	Antibody labeling Susceptible to temperature, pH Complex manufacturing process	[60,63,66]
Impedimetric sensor	Good repeatability Wide detection range	Redox is required to accelerate electron transfer Poor repeatability	[73,74,75,76,77]
FAO type	Wide detection range Easy to chemically modify Short response time Low detection limit	Specificity to be improved High detection limit Non-continuous measurement	[92,93,94,96,97,98,99,100,101,102,103]
FPOX type	Multi-sample continuous automatic analysis Good specificity	Complex manufacturing process Need mutagenesis or modification Poor oxidation activity	[104,105,106,107]
MIC type	Reusable Customizable High sensitivity	Complex manufacturing process Susceptible to impurities Non-continuous measurement Oxidation activity needs to be improved	[81,82,108,109,110,111,112,113]

## Data Availability

Not applicable.

## Abbreviation

ARS	Alizarin red s
arPES	Amine-reactive phenazine ethosulfate
AQBA	Anthraquinone boronic acid
AI	Artificial intelligence
BSA	Bovine serum albumin
CHIT	Chitosan
CMOS	Complementary metal oxide semiconductor
DM	Diabetes mellitus
DPV	Differential pulse voltammetry
ESM	Eggshell membrane
Fc	Ferrocene
Fc[CO-Cys(Trt)-OMe]_2_	Ferrocene diformylcysteine
Fc[CO-Glu-Cys-Gly-OH]	Ferrocene glutathione
FcAb	Ferrocene-tagged anti-HbA1c antibody
FIA	Flow injection analysis
FTO	Fluorine-doped tin oxide
FAO	Fructosyl amino acid oxidase
FPOX	Fructosyl peptide oxidase
FV	Fructosyl valine
FVH	Fructosyl valine histidine
GHA	GHb-targeted aptamer
GC	Glassy carbon
GCE	Glassy carbon electrode
GOx	Glucose oxidase
GA	Glutaraldehyde
HbA1c	Glycated hemoglobin
GPP	Glycated pentapeptide
AuNFs	Gold nanoflowers
AuNPs	Gold nanoparticles
GO	Graphene oxide
GS	Graphite sheet
Hp	Haptoglobin
Hb	Hemoglobin
HJNH	Heterojunction nano hybrid material
HPLC	High performance liquid chromatography
H_2_O_2_	Hydrogen peroxide
IFG	Impaired fasting glucose
IGT	Impaired glucose tolerance
ITO	Indium tin oxide
IFCC	International Federation of Clinical Chemistry
MPBA	Mercaptophenyl boronic acid
MIC	Molecularly imprinted catalyst
MIP	Molecularly imprinted polymer
NWCNT	Multiwalled carbon nanotube
Nf	Nafion
NSPC	N, S-doped porous carbon
OGTT	Oral glucose tolerance test
PtNPs	Platinum nanoparticles
PBA	Phenylboronic acid
PBS	Phosphate buffered saline
POCT	Point-of-care test
PET	Polyethylene terephthalate
PIN5COOH	Polyindole-5-carboxylic acid
pTTBA	Poly(terthiophene benzoic acid)
PVI	Polyvinyl imidazole
PAPBA	Poly(3-aminophenylboric acid)
PB	Prussian Blue
PQQ	Pyrroloquinoline quinine
RGO	Reduced graphene oxide
RVC	Reticulated vitreous carbon
SPCE	Screen-printed carbon electrode
SAM	Self-assembled monolayer
SPR	Surface plasmon resonance
SELEX	Systematic evolution of ligands by exponential enrichment
TEOS	Tetraethyl silica
T3BA	Thiophene-3-boronic acid
TiO_2_	Titanium dioxide
TBO	Toluidine blue O
Tris	Tris(hydroxymethyl) aminomethane
FDMA	1,1′-di(aminomethyl)ferrocene
MU	11-mercapto-1-undecanol
PTA	12-phosphotungstic acid
APBA	3-aminophenylboronic acid
MPA	3-mercaptopropionic acid
4-MPBA	4-mercaptophenyl boric acid
